# Antifouling Epoxy Coatings with Scots Pine Bark Extracts

**DOI:** 10.3390/molecules31010137

**Published:** 2025-12-31

**Authors:** Tomasz Szmechtyk, Magdalena Efenberger-Szmechtyk, Agata Czyżowska

**Affiliations:** 1 Department of Physical Chemistry, Faculty of Chemistry, University of Lodz, Pomorska 163/165, 90-236 Lodz, Poland; 2 Institute of Fermentation Technology & Microbiology, Faculty of Biotechnology and Food Sciences, Lodz University of Technology, Wólczańska 171/173, 90-530 Lodz, Poland; magdalena.efenberger-szmechtyk@p.lodz.pl (M.E.-S.); agata.czyzowska@p.lodz.pl (A.C.)

**Keywords:** epoxy, antifouling, coating, pine bark extract, phytochemicals

## Abstract

Antifouling coatings have to provide antibacterial performance combined with good mechanical and chemical properties. The good anticorrosive performance of tannins on steel surfaces and antibacterial activity of phytochemicals from conifers could provide a solution in the form of Scots pine bark extract. In this study, epoxy compositions with different ratios of the characterised extract (TPC, HPLC analysis of phytochemicals) were tested physically (density), mechanically (Shore D hardness, three-point bending test, Charpy impact test), chemically (DSC curing analysis, FTIR spectroscopy, chemical resistance), and microbiologically (antibacterial activity). The results were analysed and the performance of the composites was evaluated.

## 1. Introduction

Antifouling coatings play an important role in the marine industry by protecting ship hulls and submerged components of pipelines and offshore platforms from the accumulation of microorganisms, plants, algae and invertebrates. Biofouling caused by these organisms entails significant cost increases, primarily due to increased fuel consumption resulting from reduced hydrodynamic efficiency and accelerated corrosion of metallic structures.

Because the toxicity of copper- and tin-based coatings leading to increased mortality among non-target marine organisms (not involved in biofouling), new research has been conducted to find more environmentally friendly alternatives [1,2]. Also, new epoxy-based antifouling and anticorrosive coatings have been designed [3,4], containing nanomaterials or biobased additives that improve their properties.

Sun et al. [5] proposed a borneol derivative combined with glycidyl methacrylate as an antifouling agent for epoxy resin based on bisphenol A. The obtained system was partially effective against *Pseudomonas aeruginosa* and *Staphylococcus aureus*, and improvement was obtained through addition of nanosilver. A similar synergistic positive effect of both antifouling additives was observed for biofilm formation (*P. aeruginosa*) and diatom adhesion (*Pleurosigma* sp.). Gao et al. [6] synthesised an epoxy–silicone coating with imine functionalisation derived from syringaldehyde. Both antifouling and anticorrosive properties were tested. Bacteriostatic rates against *Escherichia coli* and *S. aureus* were over 99% (both) and were at least 10% higher than rates for commercial silicone (PDMS) and epoxy resins. Also, the anticorrosion properties were competitive. Liu et al. [7] used an epoxidized soybean oil (ESO) matrix cured with tannic acid (TA) to obtain anticorrosive coatings filled with graphene nanoparticles. Antimicrobial tests with *E. coli* and *S. aureus* proved the good inhibiting properties of ESO-TA coating against both, regardless of the number of graphene nanoparticles. Leaf extract of *Ixora finlaysoniana* was used as a green corrosion inhibitor for epoxy resin, achieving 80% inhibition efficiency [8]. Also, *Gliricidia sepium* leaf extract with precipitated amorphous silica in an epoxy coating provided excellent anticorrosion properties (90% inhibition efficiency) [9]. Ahmad et al. [10] proposed polyesteramide from *Pongamia glabra* oil as an anticorrosive coating without biological impact (rat-tested; no lethal dose observed).

Another way to obtain better antifouling coatings is bioinspiration. Xiong et al. [11] synthesised a copolymer of poly(*N*-vinylpyrrolidone) with antifouling properties and poly(dopamine-acrylamide) as terminal anchoring blocks imitating mussels’ adhesion capabilities. Jin et al. [12] were inspired by the antifouling strategies of Gulf parrotfish and fluorescent corals. Hydrophilic surfaces of PDMS–phosphor–silicone rubber sandwich structures with light-emitting effects were tested against bacteria (*Bacillus subtilis*, *Paracoccus pantotrophus*), the algae *Chlorella* and the diatoms *Nitzschia closterium f. minutissima*. Antifouling properties were observed only for algae and diatom representatives (samples with blue, red and yellow phosphor layers).

According to our knowledge, there are only a few studies about the application of pine products for coatings in the marine industry. Matamala et al.’s [13] complex investigation of different anticorrosive paint types (alkyd, vinyl and epoxy) included tests with pine tannins extracted from *Pinus radiata* bark as a primer layer before application of paint. Salt fog chamber and rusting tests confirmed better corrosion resistance for all three anticorrosives with pine tannin primers. Montoya et al. [14] used water–ethanolic (20:1, *v*/*v*) extracts from *Pinus radiata* bark for epoxy–polyamide anticorrosive coating. Two fractions of extracts (water-soluble WSF and water-insoluble WIF) were used separately with a polymer matrix and exposed to a salt spray chamber test. WSF, containing higher amounts of catechin and taxifolin, had better anticorrosive performance than the reference coating (epoxy–polyamide without extract). The same research group compared an epoxy system with pinus radiata bark extract with epoxy containing functionalised zinc oxide nanoparticles, but no improvements on the previous research were pointed out [15]. Perreira et al. [16], in a recent review, mentioned rosin-based (also from pine) coatings as a biocide, but without references to original papers. Extract from the bark of *Pinus sylvestris* was mixed with epoxy in our previous studies [17]. However, the antifouling properties of the composition were not under examination. Also, concentration of phytochemicals was significantly lower, and different curing chemistry conditions were applied (Mannich base instead of polyamine as a hardener).

This research is development of preliminary studies about application of Scots pine bark as biowaste. Good anticorrosive properties of pine bark phytochemicals resulting from Fe-tannins complex [13,14] can be combined with their antibacterial performance. The promising properties of early epoxy-extract composite were inspiration for the complex investigation of the chemical, mechanical and microbiological properties of epoxy systems containing extracts. Our goal was to obtain composites with extract as multifunctional additive with mixed antimicrobial and curing properties similar to other existing solutions [18,19].

## 2. Results

### 2.1. High Pressure Liquid Chromatography (HPLC) Analysis

**Figure 1:**
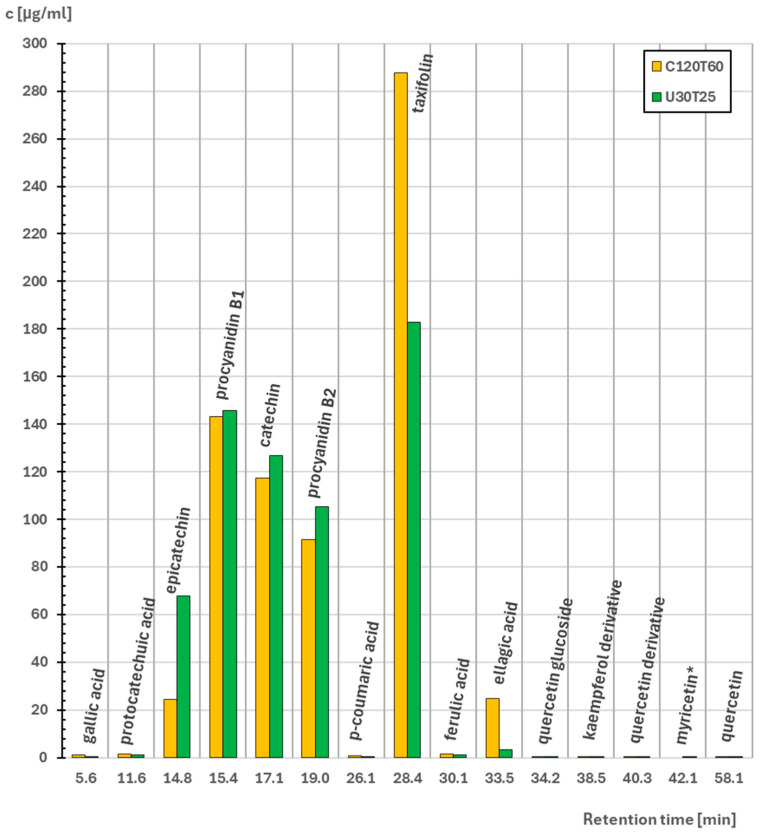
Concentrations and retention times of compounds identified by HPLC analysis. *—myricetin concentration for C120T60 extract was classified as trace amount.

The concentrations of fifteen compounds identified from extracts with their retention times were presented in Figure 1. The other eight standard phytochemicals reported in Scotch pine extracts (apigenin, caffeic acid, 4-hydroxybenzoic acid, myrcene, naringenin, pinosylvin, resveratrol and syringic acid) were determined using HPLC, but they were no present in the analysed extracts. The highest concentration was observed for taxifolin: 287.7 µg/mL (extract C120T60) and 182.9 µg/mL (extract U30T25). Also, flavan-3-ols and their derivatives were abundant in both extracts. Their concentrations in order from the highest to the lowest were: procyanidin B1 (C120T60: 143.2 µg/mL; U30T25: 145.7 µg/mL), catechin (C120T60: 117.1 µg/mL; U30T25: 126.8 µg/mL), procyanidin B2 (C120T60: 91.5 µg/mL; U30T25: 105.4 µg/mL) and epicatechin (C120T60: 24.6 µg/mL; U30T25: 67.7 µg/mL). This corresponds to reports indicating that the concentrations of these five phytochemicals increase in conifers in response to stress caused by fungal and insect attacks [20,21]. The last compound with relatively high occurrence in both extracts was ellagic acid (C120T60: 24.8 µg/mL; U30T25: 6.7 µg/mL). Concentrations of other phenolic acids (p-coumaric acid, gallic acid, ferulic acid, protocatechuic acid) and flavonols (quercetin, quercetin glucoside and quercetin derivative, kaempferol derivative, myricetin) ranged from less than 1.5 µg/mL to trace amounts.

### 2.2. Total Phenolic Content (TPC) Quantitative Analysis

The absorbance values (Abs_765_) and calculated TPC results were presented in Table 1. The highest TPC was obtained for U30T25 extract (over 2795 mg GAE per mL). Ultrasound-assisted extraction provided good results despite the short extraction time and mild conditions. The U30T25 extract was selected as the starting solution for the concentrated extract.

### 2.3. Concentrated Extract—Density, Total Solid Content and TPC

Density, total solid content (TSC), Abs_765_ and TPC of concentrated U30T25 extract were presented in Table 2. High density and TSC value (93%) confirm a significantly lower amount of methanol in extract, which was problematic in previous studies. Lower absorbance results from more diluted solution taken for UV-Vis analysis. However, the obtained TPC is twice as high as the values of the non-concentrated extract (5667.60 µg GAE/mL). It could result from phytochemicals’ losses, possibly caused by their degradation, agglomeration or limited solubility. The standard deviation level indicates that homogeneity of the initial solution was limited by the solubility of phytochemicals.

### 2.4. Preparation of Epoxy Compositions

All compositions (0–9) were examined after one week of curing at room temperature (25 °C) and air humidity of 45%. The evaluation of curing progress and calculations of percentage share and ratios (parts per hundred resin known as PHR and selective) were presented in Table 3. Almost all samples were solid after one week. Only composition 5 (100:2:10) was rejected as viscous liquid with an unsatisfactory level of curing. Five solid samples (0, 1, 2, 6 and 7) were completely cured (rigid) after one week. Another four (3, 4, 8 and 9) were partially cured (still soft and sticky) and left for another week for complete crosslinking (again at 25 °C and air humidity of 45%).

The minimum and maximum molar quantities of the initial epoxy and reactive hydrogens (*n_H_*) from amine and secondary hydroxyl groups were also summarized in Table 4. The presented molar ratios correlate with the curing results—values close to 1 and higher indicate complete curing.

### 2.5. Shore D Hardness

**Figure 2:**
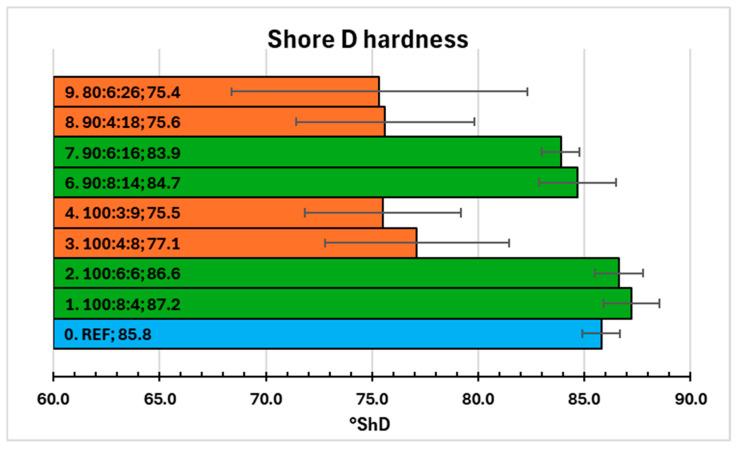
Shore D hardness results—bars with values comparable or higher than reference (blue) are marked green, orange for significantly lower values.

The Shore D hardness values of nine samples were compared in Figure 2. While the reference sample (0. REF) had almost 86°ShD, only two samples with extract slightly exceed this value (87.2°ShD and 86.6°ShD). Both (1. 100:8:4 and 2. 100:6:6) have relatively low extract content—3.57% and 5.36%, respectively. Also, compositions 6 and 7 provided good results (84.7°ShD and 83.9°ShD), comparable with reference. However, both have high amounts of extract (12.50% and 14.29%, respectively). It suggests that more important is the relatively high content of hardener, which is responsible for the high cross-linking density and the resulting hardness. This trend resembles other compositions (3, 4, 8 and 9), where a low hardener ratio gives values of °ShD less than 78. High error bars also indicate lower uniformity of these samples. Low Shore D hardnesses in combination with long curing times (over one week) were reasons for rejection of composition 4. Systems 3, 8 and 9 had similar, insufficient quality, but were left for comparison with better compositions in DSC analysis.

### 2.6. Differential Scanning Calorimetry (DSC) Analysis of Curing Process

**Figure 3:**
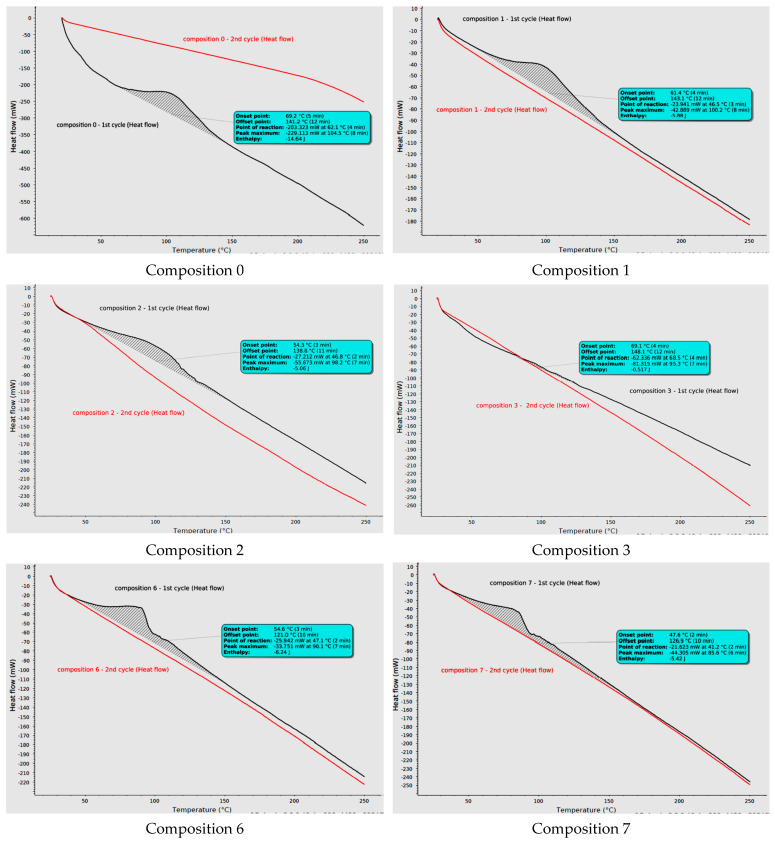

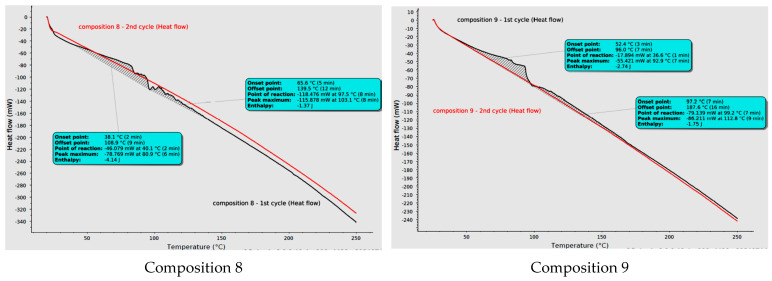
DSC thermograms of selected compositions.

The curing processes of selected epoxy systems (0, 1, 2, 3, 6, 7, 8 and 9) were compared in Figure 3. Composition 4 was not investigated as similar to 3 with worse Z-1/Epi5 ratio (slower curing). All curing reactions were exothermic, and their second cycles confirm the curing process (no peaks observed). Specific enthalpies (*h*) were calculated from enthalpies presented on diagrams divided by sample masses and expressed as joule per gram [J/g] (Table 5). The highest *h* value (−298.78 J/g) was obtained for the curing reaction of the reference composition (0). The obtained peak had a point of reaction (P_R_) at 62.1 °C, while the curing processes of most epoxy systems with extracts started between 36.6 °C and 47.1 °C. One exception is composition 3, which has P_R_ at 68.5 °C and very low specific enthalpy (only −16 J/g). This confirms that amount of amine hardener was too low (Z-1/Epi5 ratio: 0.040) and caused a sluggish reaction of this system. However, reactions of other compositions started faster than the reference thanks to the presence of extracts. It is well-known mechanism of weakening the C-O bond of the epoxy ring by hydrogen bonding from hydroxyl groups (here from phenols) [22,23,24]. The curing reaction starts at the earliest for compositions 7, 8 and 9 with the highest ex/Z-1 ratios. Another interesting regularity is the relationship between the extract PHR ratio and curing characteristics. For good compositions with extract replacing only hardener (1 and 2), only one peak is observed, and the shape of the curing curve is more similar to the reference. However, flatter slopes in the 100–150 °C region signal a splitting trend observed for systems with higher extract content (6, 7, 8 and 9). It indicates that the presence of more phenolic compounds with steric hindrance extends the duration of the second curing step after the conversion of 1° and 2° amines into 3° amines (the reaction between the epoxy rings and hydroxyl groups, also from previously opened epoxy rings). Distinct second peaks observed for epoxy systems 8 and 9 (starting at 97.5 °C and 99.2 °C, respectively) are the result of too high ex/Z-1 ratio, which causes the need for elevated temperature for better chain mobility and the chance of reaction between the epoxy ring and hydroxyl groups. Similarly to composition 3, it might also be caused by too low content of amine groups. After incorporation in epoxy matrix as 3° amines, they started acting like catalysts and their insufficient amount inhibited the epoxy-hydroxyl reaction. DSC results confirmed curing issues with systems 3, 8 and 9.

### 2.7. Preparation of Samples for Tests

**Figure 4:**
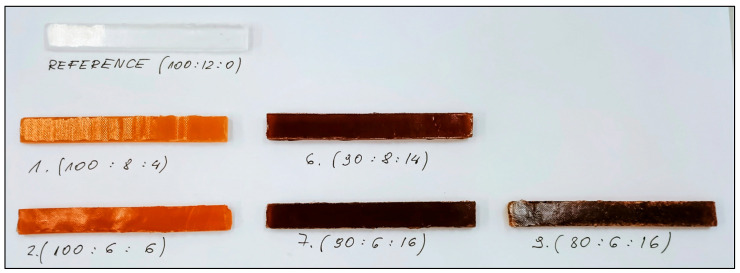
Comparison of samples for tests.

Rectangular samples of selected compositions (0, 1, 2, 6, 7 and 9) were compared together (Figure 4). Clear differences in colours were observed for samples 1 and 2, while three with extract sharing above 12% (6, 7 and 9) were more difficult to distinguish from each other. Samples of epoxy system 9 were extremely fragile, and most of them were broken during demoulding. This defect was disqualifying for both mechanical tests.

### 2.8. Density

**Figure 5:**
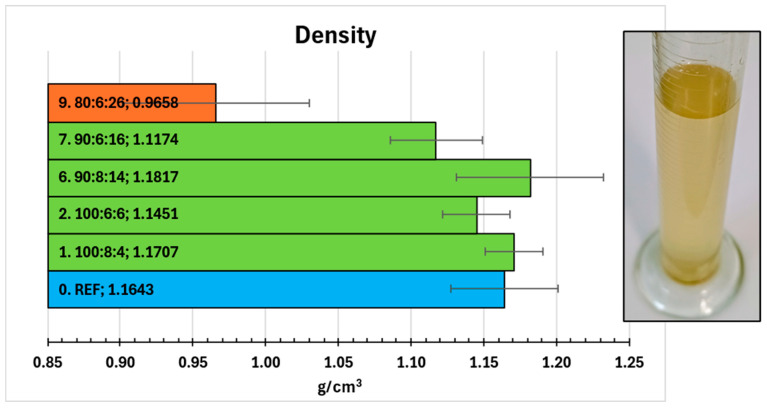
Densities of selected compositions (**left**) and color change of water from composition 9 test (**right**).

Densities of selected compositions were presented in Figure 5. Comparison with reference reveals slight differences between compositions. Higher content of amine hardener (1. 100:8:4 and 6. 90:8:14) corresponds to higher density. It is expected correlation due to the well-known, positive effect of hardener on crosslink density. The increase of crosslink density causes increase of composite density (more packed atoms per volume) [25,26,27]. Anyway, densities of aforementioned two composites surpass 0. REF result, which has a higher Z-1/Epi5 ratio (12:100 = 0.120) than composites 1 (8:100 = 0.080) and 6 (8:90 = 0.089). It could result from the positive effect of functional (mainly hydroxyl) groups of phytochemicals from extract. Still the presence of amine groups is necessary, what can be observed for composites 2 (100:6:6) and 7 (90:6:16). Their less dense structure could be associated with too low content of amine groups (Z-1/Epi5 ratios are 0.060 and 0.067, respectively). Composition 9 showed the lowest density of tested systems (0.9658 g/cm^3^). Being slightly less dense than water caused samples’ floating on the surface (they were gently pushed down to stay under the surface). Also, the highest standard deviation (±0.0643 g/cm^3^) suggests that 26 PHR of extract caused increased porosity and inhomogeneity of samples. These issues could result from limited solubility of phytochemicals in epoxy resin and agglomeration of insoluble particles, which did not react with epoxy matrix. The water-soluble fraction of these compounds was rinsed out during the test and caused yellowing of the water in the measuring cylinder (Figure 5).

### 2.9. Antibacterial Activity

The R (reduction rate) values of selected epoxy systems and tested bacteria species were presented in Table 6. Negative R values represent promoting effect on bacteria growth, while positive R indicates growth-inhibiting properties of composite. Composite 0, as commercial epoxy system without extract was a reference point.

Composite 9 containing the highest concentration of the extract, revealed the strongest antibacterial properties with all R values above 3. However, such composite should be very carefully introduced as an antifouling coating. The release of phytochemicals with potent antibacterial activity may cause side effects in the marine environment similar to those observed with coatings containing copper and organotin compounds. Also, other properties of composite 9 will hinder its application as coating (low hardness, fragility, curing issues).

Composite 0 did not show antibacterial activity towards *S. aureus*, *B. subtilis* and *K. aerogenes*. Surprisingly, it significantly inhibited growth (R = 5.00) of *E. faecium*. It could result from initial enzymatic degradation of epoxy by bacteria [28,29,30] and release of products toxic for *E. faecium* (oligomers with functional groups). This may lead to autolytic process of the strain. Peptidoglycans and teichoic acids from bacteria cell walls can act as endotoxins and contribute to resin degradation [30,31]. These processes might still lead to further degradation of epoxy resin in the future, followed by more extensive biofouling. Partially degraded coating could be more susceptible to attack of other types of biofouling organisms. Composites 1, 2 and 6 revealed weaker antibacterial properties towards *E. faecium* compared to composite 0. This might be due to slower initial enzymatic degradation provided by the presence of extracts and can be beneficial in combating further stages of biofouling. However, these mechanisms are rather complex and need further investigation with other methods considering long-term biodegradation process.

Antibacterial activity against *E. coli* strain was not observed for most samples (system 9 as the only exception). On the other hand, the promoting effect was significantly lower for compositions 2, 6 and 7. This indicates that above 4 PHR, the presence of phytochemicals in the matrix hinders *E. coli* activity. Another representative of Gram-negative *Enterobacteriaceae*—*K. aerogenes* also grew well on epoxy resin. However, in contrast to *E. coli* the systems with extracts did not diminish its promoting effect. The increasing number of *E. coli* and *K. aerogenes* on epoxy resin could be caused by using the polymer matrix as a carbon source. Literature data report that some bacteria strains can participate in the biodegradation process of epoxy resin [28,29].

Epoxy systems 1 and 2 did not have the bactericidal effect of *S. aureus*. However, compositions with higher extract content (6 and 7, with 14 PHR and 16 PHR, respectively) provided mild bacteria inhibition. Composition 6 was slightly better than 7. It could result from its higher density (also better crosslink density). A similar tendency was observed for *B. subtilis* results. The number of bacteria on both systems with higher density (1 and 6) was reduced (R values were 0.60 and 0.83, respectively). Lower density resulted in a lower antibacterial effect (7: R = 0.30) and combined with low extract content (2), even in a negative R value (−0.35).

### 2.10. Fourier Transform Infrared (FTIR) Analysis of Composites

**Figure 6:**
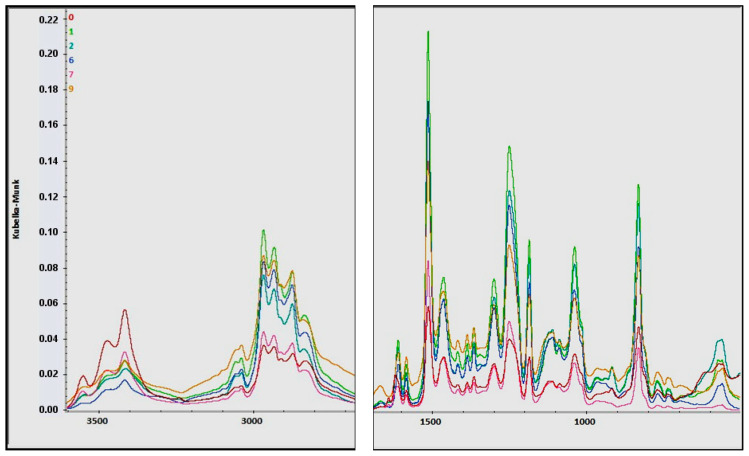
Comparison of FTIR spectra (composites: 0, 1, 2, 6, 7 and 9)—wavelength ranges from 3600 cm^−1^ to 2700 cm^−1^ (**left**) and from 1700 cm^−1^ to 500 cm^−1^ (**right**).

FTIR spectra of composites were compared in Figure 6. Areas of the spectra from 2700 cm^−1^ to 1700 cm^−1^ did not provide important peaks and were removed. Three peaks at about 3550 cm^−1^, 3470 cm^−1^ and 3415 cm^−1^ (stretching vibrations of amine groups and hydroxyl groups, both free and associated) [32,33,34] show significantly greater intensity for reference than for compositions with extract. Lower concentration of these groups in compositions with extract could be a result of presence of aromatic compounds with less hydroxyl groups and without amine groups. The opposite situation was observed for bands of area 3100–2800 cm^−1^. Both C-H bonds of saturated C-C bonds and unsaturated C=C bonds from aromatic compounds have better presence in compositions with extract. Again, various phytochemicals provided these bonds. Peaks around 3035 cm^−1^, 2965 cm^−1^, 2930 cm^−1^ and 2870 cm^−1^ are sharper (compared to peak at 2830 cm^−1^) for no-reference compositions. This results from assignment of peak at 2830 cm^−1^ to stretching vibrations of methylene groups in α-position to amine (mainly in tertiary amines) [35,36]. The lack of strong peaks around 1700 cm^−1^ indicates that all composites have no C=O groups from most of aliphatic moieties: aldehydes, ketones, esters, or carboxylic acids. Higher intensity of the band around 1650 cm^−1^ for composites with extract (especially system 9) indicates the presence of a carbonyl group from heterocyclic ring C of flavanonol (mainly taxifolin). A strong peak around 1510 cm^−1^ is from C=C bonds of aromatic compounds (higher intensities for compositions with extracts, a similar situation to C-H region). Two peaks around 1250 cm^−1^ and 1050 cm^−1^ indicate higher concentration of alkyl-aryl ether for most of composites with extract (increased by products of reaction between epoxy ring and phenolic compounds).

### 2.11. Adhesion Tests

The scale-based evaluation of both adhesion tests with representative pictures of samples from test was presented in Table 7. All results had good repeatability, as the three samples for each composition-test combination exhibited similar levels of surface damage. Reference samples achieved best rating (0) in the x-cut test and a very good rating (1) in the cross-cut test; small flakes were observed at the intersections, but less than 5% of the tested area was affected.

Samples of composition 1 achieved the best ratings (0) in both tests, indicating that a 4 PHR ratio of extract provided improved bonding of 180 µm thin coating to steel. In contrast, replacing part of amine hardener with a higher amount of extract (composition 2) resulted in slightly poorer adhesion in both tests (ratings of 1 in each case). Similarly, compositions with a 90 PHR ratio of epoxy (6 and 7) had even worse bonding for 180 µm thin coating, with both achieving a rating of 2 (affected area between 5 and 15%). 

A higher PHR ratio of amine hardener (8 PHR) still resulted in a rating of 0 in the x-cut test for composition 6, whereas lower hardener content (6 PHR) in composition 7 led to worse x-cut rating of 1. Additionally, both compositions with a 90 PHR ratio of epoxy showed slight surface colour inhomogeneity, attributed to the higher tannin content from the extract. Greater colour inhomogeneity was observed for system 9, particularly in the x-cut test and for thicker coating. However, overall inhomogeneity more strongly affected the 180 µm thin coating, which received a rating of 2 in the cross-cut adhesion test. Although, this rating represents an average between ratings 1 and 3, as most test squares had unaffected edges (rating 1), while only a few exhibited detached large flakes (rating 3).

### 2.12. Charpy Impact Test

**Figure 7:**
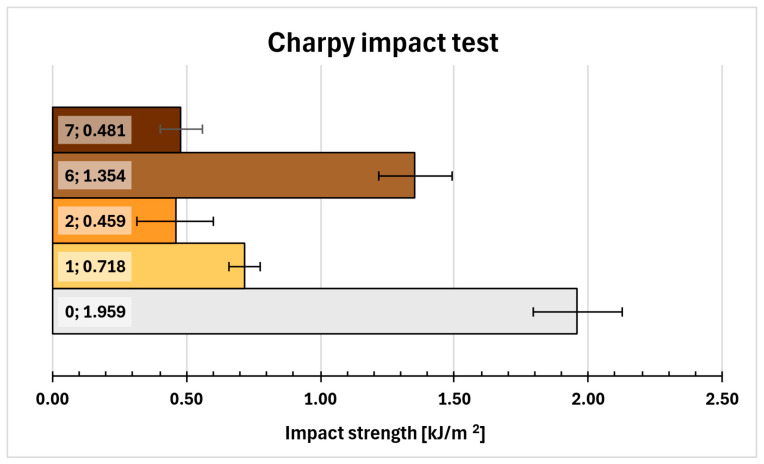
Charpy impact test results.

The impact strength results were compared in Figure 7. The reference sample (0) had the highest mean impact strength (1.959 kJ/m^2^), followed by compositions 6 (1.354 kJ/m^2^) and 1 (0.718 kJ/m^2^). In contrast, compositions 2 and 7 did not exceed 0.5 kJ/m^2^.

A clear correlation between Z-1/Epi5 ratio and impact strength was observed with impact strength decreasing as the ratio decreased: 0 (0.120), 6 (0.089), 1 (0.080), 7 (0.067), 2 (0.060).

### 2.13. Three-Point Bending Test

**Figure 8:**
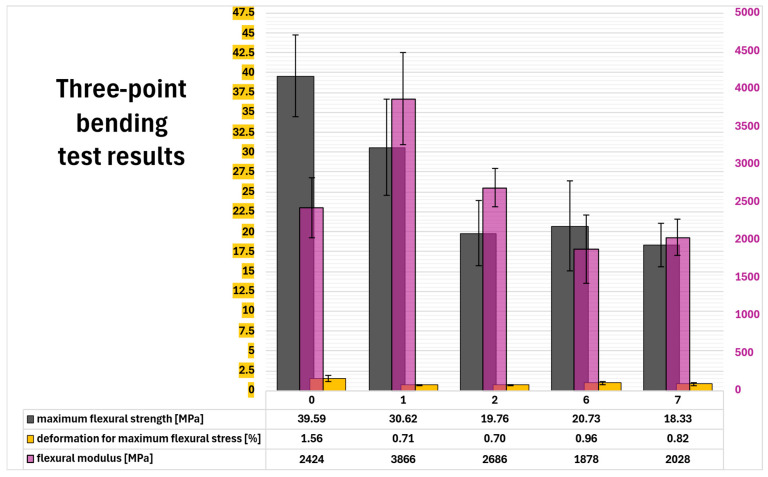
Three-point bending test results. Left vertical axis corresponds with maximum flexural strength (dark grey columns) and deformation for maximum flexural stress (yellow columns). Right vertical axis shows values for flexural modulus (violet columns).

Three-point bending test provided complex results (Figure 8) about composite maximum flexural strength (σ_fM_). Reference composition (0) had highest σ_fM_ (almost 40 MPa), but plastic deformation (ε_fM_ = 1.56%) played a significant role. In contrast to the second-best composition (1. σ_fM_ = 30.62 MPa), which was largely due to the elastic component (the highest flexural modulus: Ef = 3866 MPa). Composition 2 had similar ε_fM_ (0.70%) to composition 1, but with Ef slightly above reference (2686 MPa compared to 2424 MPa) showed significantly lower flexural strength (19.76 MPa). Composition 6, with the lowest Ef (1878 MPa) and second best ε_fM_ value (0.96%), had σ_fM_ = 20.73 MPa. Last composition (7), with highest amount of extract had also the lowest flexural strength (18.33 MPa). This was result of average plastic component (ε_fM_ = 0.82%) and low elastic component (E_f_ = 2028 MPa).

### 2.14. Chemical Resistance

**Figure 9:**
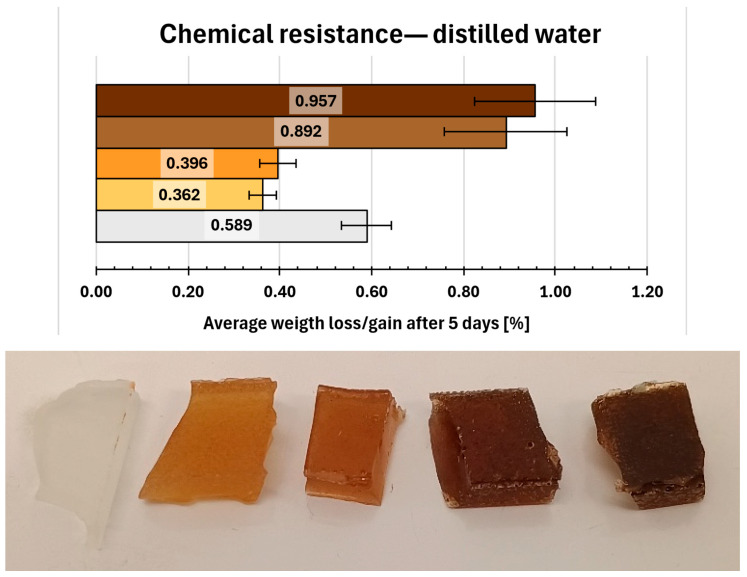
Gravimetric and visual examination of composite representative samples after immersion in distilled water. Samples are marked on the graph with colours resembling their real hue. From bottom to top (graph) and from left to right (photo): 0—white, 1—yellow, 2—orange, 6—light brown, 7—dark brown.

**Figure 10:**
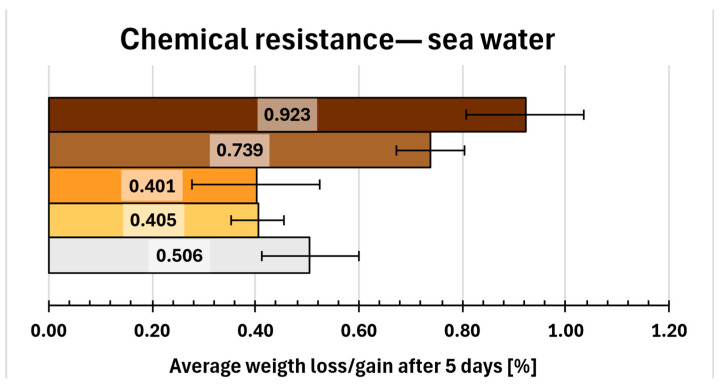

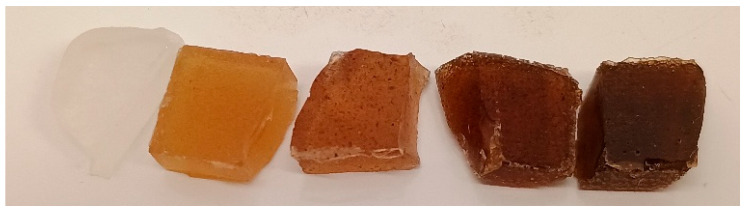
Gravimetric and visual examination of composite representative samples after immersion in sea water. Samples are marked on the graph with colours resembling their real hue. From bottom to top (graph) and from left to right (photo): 0—white, 1—yellow, 2—orange, 6—light brown, 7—dark brown.

**Figure 11:**
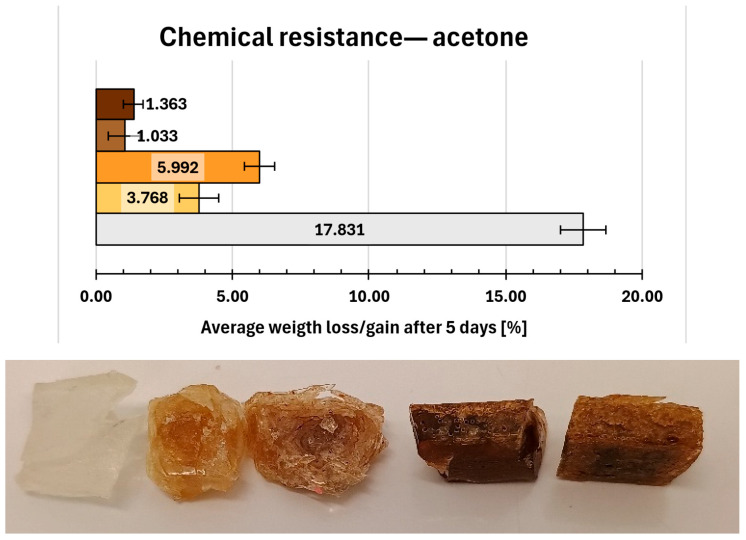
Gravimetric and visual examination of composite representative samples after immersion in acetone. Samples are marked on the graph with colours resembling their real hue. From bottom to top (graph) and from left to right (photo): 0—white, 1—yellow, 2—orange, 6—light brown, 7—dark brown.

**Figure 12:**
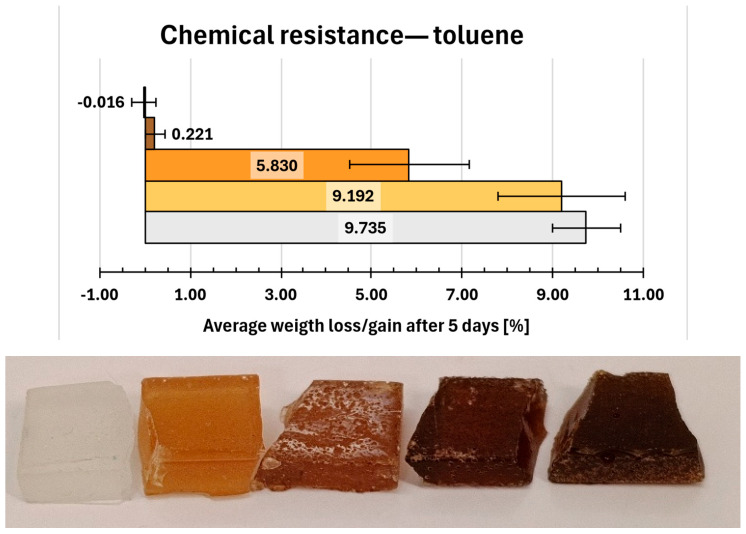
Gravimetric and visual examination of composite representative samples after immersion in toluene. Samples are marked on the graph with colours resembling their real hue. From bottom to top (graph) and from left to right (photo): 0—white, 1—yellow, 2—orange, 6—light brown, 7—dark brown.

**Figure 13:**
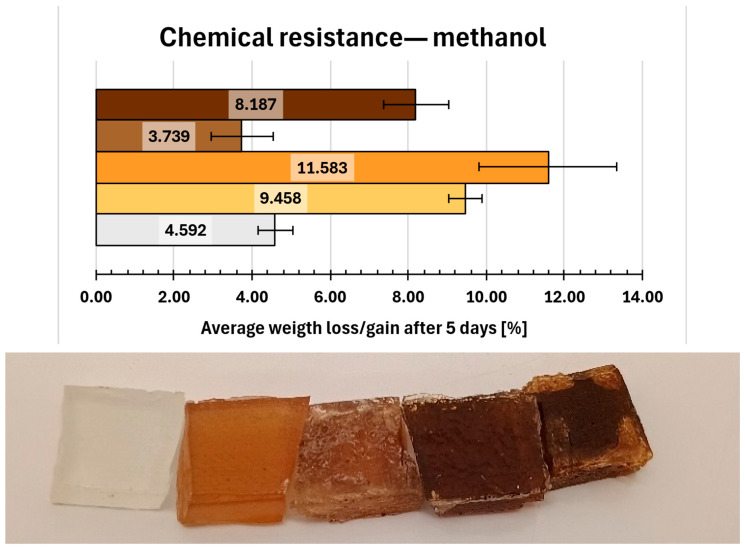
Gravimetric and visual examination of composite representative samples after immersion in methanol. Samples are marked on the graph with colours resembling their real hue. From bottom to top (graph) and from left to right (photo): 0—white, 1—yellow, 2—orange, 6—light brown, 7—dark brown.

**Figure 14:**
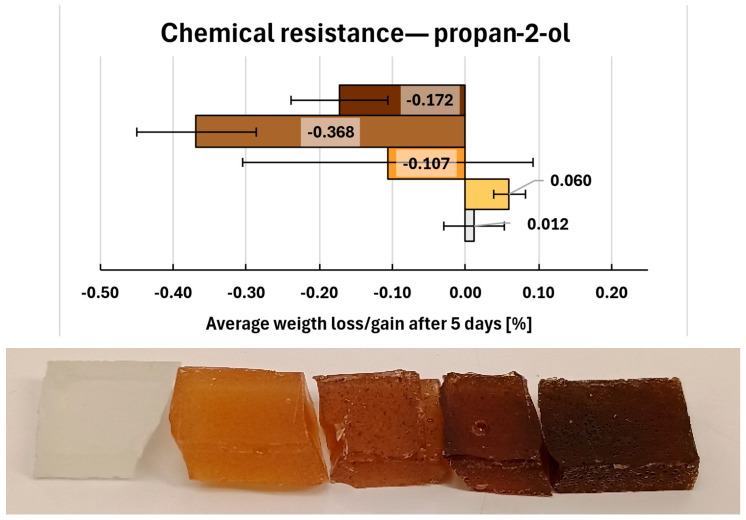
Gravimetric and visual examination of composite representative samples after immersion in propan-2-ol. Samples are marked on the graph with colours resembling their real hue. From bottom to top (graph) and from left to right (photo): 0—white, 1—yellow, 2—orange, 6—light brown, 7—dark brown.

**Figure 15:**
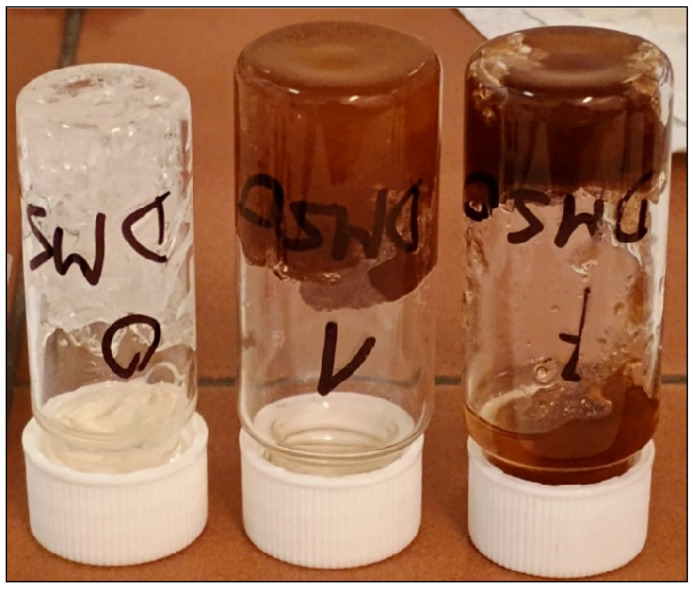
Selected samples after 5-day immersion in DMSO. Solvent absorbed by samples caused their clustering.

The results of the chemical resistance tests were presented in Figure 9, Figure 10, Figure 11, Figure 12, Figure 13, Figure 14 and Figure 15. The distilled water (Figure 9) as a reference non-aggressive solvent, yielded a mean weight gain of only 0.589% for the reference samples (0. REF), indicating high chemical resistance. The remaining compositions exhibited comparable behaviour, with weight gains below 1% and no visible degradation of samples observed.

A slight improvement in chemical resistance (compared to 0. REF composition) was noted for compositions 1 and 2, both of which showed average weight gains under 0.4%. On the other hand, compositions 6 and 7 approached 1% threshold, with weight gains of 0.892% and 0.957% respectively. All observed mass increases are most likely attributable to water adsorption within surfacemicrodefects generated during prior mechanical testing.

A similar trend was observed for samples immersed in sea water (Figure 10). Compositions 1 and 2 again demonstrated slightly better chemical resistance than the reference, with average weight gains of approximately 0.4%, compared to 0.5% for the reference. In contrast, composition 7 exhibited higher weight gain, exceeding 0.9% (0.923%). Only composition 6 showed slightly better results in sea water (0.739%) compared to those in distilled water (0.892%).

Acetone, used as more aggressive solvent (Figure 11) caused pronounced degradation and significantly higher weight gain, particularly for the reference composition (17.8%). Also, major cleavages of 0.REF samples were observed. Composites 1 and 2 exhibited lower weight increase (3.768% and 5.992%, respectively) accompanied by visible surface degradation manifested as surface dullness and detachment of small fragments.

Acceptable chemical resistance in acetone was observed only for composites 6 and 7. The composites showed weight gains slight above 1% and exhibited less severe degradation, limited primarily to the surfaces of existing fractures.

Immersion in toluene (Figure 12) also resulted in significant swelling of compositions 0, 1 and 2 samples (9.735%, 9.192% and 5.830%, respectively). However, visible surface dullness was observed only for composition 2. In contrast, compositions 6 and 7 exhibited high chemical resistance to toluene, with 0.221% weight gain (6) and 0.016% weight loss (7); neither displayed any visible surface degradation.

Figure 13 presents the results of immersion in methanol. Only composition 6 exhibited lower swelling than the reference sample (weight gain of 3.739% compared to 4.592% for the reference). This improved resistance may be attributed to the combined effect of relatively high amine/epoxy ratio (0.089) and elevated phytochemical content. All other compositions showed weight increases of approximately 10%. Visible changes, primarily surface dullness, were observed for samples 2 and 7.

Immersion in propan-2-ol (Figure 14) did not cause observable damage to any of the tested compositions. Only minor mass changes were recorded-small weight gains: 0.012% for 0. REF and 0.060% for composite 1,as well as slightly higher weight losses for remaining compositions (ranging from −0.107% to −0.368%). No visible changes in sample appearance were detected.

Exposure to DMSO resulted in severe damage to all samples. The extent of swelling was too excessive and could not be reliably quantified by gravimetric measurements (Figure 15).

## 3. Discussion

Presented preliminary tests provided comprehensive information about the extracts and obtained composites. The selected extract contained high levels of phenolic compounds (taxifolin, catechin, epicatechin, procyanidins) with five or more accessible hydroxyl groups per molecule. These functional groups can promote a more branched and dense resin network. The TPC value of the concentrated extract confirmed this high availability of hydroxyl groups (5.6 mg GAE/mL).

Incorporation of this extract into epoxy compositions at different ratios produced a wide range of material properties. The best-performing samples exhibited enhaced Shore D hardness, higher cross-link density and nearly comparable mechanical performance to the reference. Additionally, an autocatalytic effect of phenolic compounds on epoxy-amine reactions was observed across all compositions. However, the ratios of each mixture component were crucial. Low amine content limited the reactions between epoxy and hydroxyl groups. Too high concentration of phenolic compounds causes inhibition of epoxy-hydroxyl reaction. Moreover, composition 9 with high ex/Epi5 ratio had limited cross-linking capabilities due to poor solubility of phytochemicals in epoxy matrix. This led to visible leaching of these compounds into water, which provided antimicrobial activity of phytochemicals against all tested Gram-positive and Gram-negative strains of bacteria. Other epoxy systems demonstrated more selective antibacterial effects, limited by immobilization of phenolic moieties in polymer matrix. Notably, chain termination by polyphenols still provided effective inhibition of G-positive bacteria and reduced E. coli growth compared to reference composition. 

High extract content also influenced chemical resistance. Compositions with high extract PHR ratio showed significantly improved resistance to toluene and acetone, but exhibited reduced methanol resistance in most samples.

Overall, two epoxy systems displayed particularly promising properties. Composition 1 (100:8:4) showed properties most similar to the reference, with improved hardness, stronger adhesion as coating, slightly better water and acetone resistance, and antimicrobial activity, making it a viable alternative to commercial epoxy systems. Composition 6 (90:8:14) with increased extract content had the highest density among the tested samples, good impact strength and excellent adhesion for thicker layers. Coupled with very good chemical resistance (toluene, acetone, and notably, methanol) and superior antimicrobial properties among the stable solid samples, composition 6 could serve effectively as an antifouling coating for specific applications.

The results also indicate directions of further improvement. Designing composites with reduced phytochemicals leaching—minimizing environmental impact while maintaining mechanical and chemical performance—(could be our further goal. More complex and controllable systems of phytochemicals release (e.g., microcapsules [37]) could offer a promising route to novel antifouling coatings. Although, such systems can provide leakage of more concentrated antibacterial mixtures, mechanism of release could be problematic in demanding water environment. Additionally, exploring extracts from other trees species could provide more targeted mixtures of polyphenols with complementary or enhanced antifouling efficiency.

## 4. Materials and Methods

### 4.1. Materials

Methanol was purchased in two purities: HPLC grade (>99.9%; Honeywell, Charlotte, NC, USA) for UV-Vis and HPLC analysis and reagent grade (99.8%; Avantor Performance Materials Poland S.A., Gliwice, Poland) for distillation of larger quantities and chemical resistance tests. Reagent grade: dimethyl sulfoxide (99.7%), propan-2-ol (99.7%) and toluene (99.5%) were obtained from Avantor Performance Materials Poland S.A. Acetone (reagent grade, 99.5%), Folin-Ciocalteu’s reagent and sodium carbonate (anhydrous, 99.8%) were purchased from Chempur (Piekary Śląskie, Poland). Epoxy resin Epidian 5 and amine hardener Z-1 were produced by Sarzyna Chemical (Nowa Sarzyna, Poland). Sodium chloride (>99%, ACS reagent grade) was purchased from Sigma-Aldrich (St. Luis, MO, USA). All HPLC standards were obtained from Merck KGaA (Darmstadt, Germany) at HPLC grade or highest available instead.

### 4.2. Scots Pine Bark Grinding

Bark was collected from different fallen trees of Scots pine (*Pinus sylvestris*) around Łódź, Poland. Bark flakes were crushed by hand and ground using Davide 4 V 550 W mill (Novital, Lonate Pozzolo, Italy) equipped with 1 mm sieve. The excess of ground bark was stored in refrigerator.

### 4.3. Extraction for Qualitative and Quantitative Analysis

Extraction from bark was performed under three different conditions (Table 8). 10 mL of methanol (HPLC grade) was poured into each flask containing 1 g of ground bark. Conventional extraction was performed with Heidolph MR-Hei Standard hot plate (Heidolph Instruments, Schwabach, Germany) with magnetic stirrer (750 rpm). For ultrasound assisted extraction, Bandelin Sonorex DL 510 H (Bandelin Electronics, Berlin, Germany) ultrasonic bath was used (ultrasonic peak power: 640 W). The sediment was separated by centrifugation. Obtained clear extracts were stored in refrigerator.

### 4.4. High Pressure Liquid Chromatography (HPLC)

C120T60 and U30T25 extracts were selected for HPLC analysis as representatives of both types of extraction. Freshly prepared extracts were filtered through 0.45 µm membrane before injection into Finnigan Surveyor HPLC system (Thermo Fisher Scientific Inc., Waltham, MA, USA). Chromatographic separation was performed under temperature 40 °C, using Spherisorb ODS2 column (80 Å, 5 µm, 4.6 × 250 mm) from Waters Corporation (Milford, MA, USA). Regime of separation (flow, percentage composition changes of mobile phases, time of change) was presented in Table 9. Photodiode array (PDA) and fluorescence (FL) detectors were used for identification and quantification of phytochemicals. Three wavelengths (280 nm, 320 nm and 360 nm) were selected for detection with the use of PDA. For FL, wavelength of excitation was 250 nm and wavelength of emission—350 nm.

### 4.5. Total Phenolic Content Quantitative Analysis

Folin-Ciocalteu method [38] was selected for total phenolic content (TPC) measurement of bark extracts. Distilled water was mixed with each extract (19:1, *v*:*v*). 100 µL of obtained solutions was taken. 200 µL of Folin-Ciocalteu reagent and 3000 µL of sodium carbonate solution (6.67%) were added to each extract solution and mixed. All samples were stored in dark place (1 h) and their absorbance (at λ = 765 nm) was measured using Specord 50 UV-Vis spectrophotometer (Analytik Jena AG, Jena, Germany). WinAspect 2.3.1.0. software (Analytik Jena AG, Jena, Germany) was used. The absorbance of each extract was collected and averaged from six samples. The calibration curve (absorbance = f(mass of gallic acid)) was used for calculating TPC values. TPC values were expressed as micrograms of gallic acid equivalent (GAE) per millilitre.

### 4.6. Concentrated Extract—Preparation

One extract was selected as presenting highest TPC value among others and competitive composition of phytochemicals. Five flasks with the same bark/methanol ratio (20 g per 200 mL) were prepared and selected extraction procedure was performed. Due to the larger volume of the extract, centrifugation was replaced by sieving (sieve opening: 1 mm). Obtained extract without large residues was concentrated using SBS-RV-2000 rotary evaporator (Steinberg Systems expondo GmbH, Berlin, Germany). All methanol was evaporated under mild conditions (40 °C, 60 rpm, 25’’Hg vacuum) and remaining solid content was collected with 100 mL of clean methanol. Procedure was repeated several times to obtain enough concentrated extract.

### 4.7. Concentrated Extract—Density, Total Solid Content and TPC

The concentrated extract was characterised by density, total solid content (TSC) and TPC. Density and TSC were measured using automatic pipette (HTL Labmate Pro LMP1000, Corning HTL SA, Warsaw, Poland) and Sartorius RC210D balance (Sartorius, Göttingen, Germany) with an accuracy of 10^−5^ g. Six portions (1 mL each) of freshly mixed extract were collected into weighting bottles with lids. The weighting bottles with extracts were weighted immediately (tare weight was also considered). Density was calculated (d = m/V) and opened bottles were left under fume hood for 72 h. All samples were weighted again after evaporation of volatile compounds (mainly methanol). TSC was calculated as ratio of solid residues’ mass after drying to extract’s mass before drying, expressed as percent. Absorbance (at λ = 765 nm) of six samples of concentrated extract was collected as for previous extract (only dilution was 1:51 instead of 1:20). Also, TPC was calculated using same calibration curve (dilution recalculated). All obtained extracts were stored in refrigerator before further use.

### 4.8. Preparation of Epoxy Compositions

Epoxy compositions were obtained by mixing in different ratios: Epidian 5 epoxy resin (diglycidyl ether of bisphenol A; viscosity @25 °C—20,000–30,000 mPa∙s; density @20 °C—1.18–1.19 g/cm^3^; epoxy value—0.48–0.51 mol per 100 g) with Z-1 amine hardener (triethylenetetramine; viscosity @25 °C—20–30 mPa∙s; density @20 °C—0.98 g/cm^3^; amine value—at least 1100 mg KOH/g) and concentrated U30T25 extract (Table 10). Components ratios were expressed as parts per hundred resin (PHR). However, systems from 6 to 9 had resin PHR value less than 100. It resulted from the fact that missing PHR of resin was replaced with extract (instead of replacing hardener with extract). Epidian 5 was mixed with the extract in flat polypropylene container and then Z-1 was added. For reference (REF) composition (No. 0) no extract was used. Each mixture was left for curing in the container at room temperature (25 °C) and air humidity of 45%. All compositions were examined after 1 week. Uncured, liquid compositions were rejected.

Minimum and maximum molar quantities of initial epoxy and hydrogens (n_H_) of amine and secondary hydroxyl groups were calculated. Epoxy groups were calculated from epoxy value of Epidian 5 resin, provided by manufacturer (0.480–0.515 mol/100 g of resin), taking into account lower epoxy PHR ratio of composites from 6 to 9. Minimum molar quantities of amine groups were calculated from min. amine value (AV) also provided by manufacturer (1100 mg KOH/g). Theoretical number of nitrogen atoms (n_N_) per compound was calculated from Equation (1):

AV = (n_N_ × 56.1 × 1000)/M_TETA_ (1)

where M_TETA_ is molar mass of triethylenetetramine (TETA) and equals 146.238 g/mol. Obtained minimum theoretical number of nitrogen per compound was only 2.87, meaning that Z-1 hardener could contain TETA and impurities without nitrogen atoms (or low AV was intentional misleading by the manufacturer). Maximum n_N_ was 4 (as real number of amine groups in TETA, assuming that Z-1 contains only TETA) and number of hydrogens (n_H_) in single TETA molecule was 6. This gives nitrogen to hydrogen ratio 0.67 and allows to calculate minimum theoretical number of hydrogen for n_N_ = 2.87, which is 4.31. Numbers of TETA moles were calculated from PHR ratio [g] divided by M_TETA_ and multiplied by both n_H_ (min. and max.) to give molar quantities of n_H_ of amine groups. n_H_ in both amine and secondary hydroxyl groups was calculated by multiplying by 2.

### 4.9. Shore D Hardness

Shore D hardness of the samples that passed the curing criterion was measured at nine points for each. Test was conducted with Sauter HBD 100-0 Shore D hardness tester (Sauter, Freiburg, Germany). Tester accuracy was 1 Shore hardness degree (°ShD). Obtained results were averaged.

### 4.10. Differential Scanning Calorimetry (DSC) Analysis of Curing Process

The curing processes of selected compositions were investigated using Linseis Chip-DSC 100 calorimeter (Linseis Messgeraete GmbH, Selb, Germany) with respective Linseis software. 22.4 g of each composition was mixed in plastic container (mixing time: 5 min) and about 30 mg of mixture was placed in 100 µL aluminium crucible with pierced lid. All samples were heated twice from 25 °C to 250 °C (heating speed: 10 °C/min). Time of each sample preparation (from end of mixing to start of DSC analysis) was less than 5 min.

### 4.11. Preparation of Samples for Tests

Selected compositions were poured into silicone moulds to obtain 18 rectangular samples (80 mm × 10 mm × 4 mm) for each composition and degassed using vibrating table (10 min). Samples were demoulded after two weeks (curing at 25 °C) and overflowed edges were removed. Prepared samples were stored in room conditions (temperature: 23 °C, air humidity: 45%) before mechanical tests.

### 4.12. Density

Densities of selected samples were calculated from their mass to volume ratios (each averaged from 9 results). Masses were collected using Sartorius RC210D balance (Sartorius, Göttingen, Germany) with an accuracy of 10^−5^ g. Volumes of samples were obtained as a volume change caused by immersion of sample in measuring cylinder (accuracy of 0.5 mL) with distilled water.

### 4.13. Antibacterial Activity

The antimicrobial activity of selected composites was tested according to the ASTM E2180 standard [39] (Standard Test Method for Determining the Activity of Incorporated Antimicrobial Agent(s) In Polymeric or Hydrophobic Materials). Bacteria strains typical for water environment were used in this study. They were obtained from the American Type Culture Collection (*Escherichia coli* ATCC 10536, *Staphylococcus aureus* ATCC 6538, *Bacillus subtilis* ATCC 6633), Polish Culture of Microorganisms (*Klebsiella aerogenes* PCM 532) and one was isolated from water (*Enterococcus faecium* WR1, accession number in Gen Bank MG911720). The microbial cultures were stored on TSA (Tryptic Soy Agar, from Merck KGaA, Darmstadt, Germany) medium and activated before the experiment.

The tested epoxy resins were cut into squares approximately 2 × 2 cm. They were disinfected with ethanol and rinsed with sterile distilled water to remove residual alcohol. The materials were placed in a sterile vessel. The suspensions of the tested microorganisms of approximately 10^6^ CFU/mL (CFU—colony-forming unit) were prepared in physiological saline with 0.3% agar added. Next, bacterial suspension (0.2 mL) was spread on the surface of polymer composites. Determining microbial counts was performed immediately after application of the suspension (t = 0) and after 24 h of incubation (t = 24 h). The culture method on TSA medium was used, and the plates were incubated at 30 °C for 24–48 h. The bacteria colonies grown were counted and the results were expressed as the number of colony-forming units per cm^2^ of the material surface (CFU/cm^2^). The reduction rate (R) of microorganisms was calculated according to the following Formula (2):

R = (log CFU/cm^2^_t=0_ − log CFU/cm^2^_t=24h_) (2)

where both CFU/cm^2^_t=0_ and CFU/cm^2^_t=24h_ mean number of microbial cells per surface for t = 0 and t = 24 h, respectively.

### 4.14. Fourier Transform Infrared (FTIR) Analysis of Composites

Selected composites were powdered, mixed with KBr and analysed using Nicolet iS50 FTIR spectrometer (Thermo Fisher Scientific Inc., Waltham, MA, USA) with an EasiDiff reflection accessory (Pike Technologies, Madison, WI, USA). Scans were collected in the spectral range of 3650–500 cm^−1^ using mercury cadmium telluride detector (32 scans per sample, 8 cm^−1^ optical resolution). Obtained spectra were converted using Kubelka-Munk correction and compared using OMNIC 9.11.727 software (Thermo Fisher Scientific, Waltham, MA, USA).

### 4.15. Adhesion Tests

The adhesive strength of selected epoxy compositions as coatings on stainless steel plates was examined using x-cut adhesion test (according to ISO 16276-2 standard [40]) and cross-cut adhesion test (according to ISO 2409 standard [41]). Coating thickness for cross-cut test was 180 µm and was obtained by using TQC Sheen Master Paint Plate (Industrial Physics, New Albany, IN, USA). TestAn paralell knife with 3 mm grid (Anticorr, Gdańsk, Poland) was used to create cross-cut area. Coating thickness for x-cut was above 250 µm. All samples were conditioned before tests at 23 °C, in air humidity 45%. The same conditions were during tests. The evaluation of results (from 3 different places of each sample) was conducted based on 6-point scales (from 0 to 5, where 0 means no peeling or flaking and 5 means complete detachment of coating).

### 4.16. Charpy Impact Test and Three-Point Bending Test

Charpy impact test was performed using Cometech QC-639P/Q universal impact tester (Cometech, Taichung, Taiwan) with 2.9 m/s striking speed of Charpy vise equipped with 2J pendulum hammer. Three-point bending test was performed using Zwick/Roell 1435 universal testing machine (Zwick/Roell, Ulm, Germany) The testing speed was 2 mm/min. Results from nine rectangular samples (80 mm × 10 mm × 4 mm) of selected epoxy systems were obtained for each test-system combination and averaged.

### 4.17. Chemical Resistance

Shards of samples from mechanical tests were used for chemical resistance analysis—5-day immersion in selected solvents: distilled water, sea water, acetone, toluene, methanol, propan-2-ol and dimethyl sulfoxide (DMSO). Sea water was prepared by dissolving sodium chloride (3.5 g) in 100 mL measuring flask and diluted up to the line with distilled water. Shards were weighted before and after immersion (dried gently with towel paper and left for 24 h under fume hood) with accuracy of 10^−5^ g, using Sartorius RC210D balance (Sartorius, Göttingen, Germany). Results (percentage weight loss or gain) were averaged from six samples for each composite-solvent combination.

## 5. Conclusions

Results of preliminary tests presented in this article are valuable for further research. Epoxy compositions with Scots pine bark extracts are interesting alternatives to commercially available epoxy resins. Thanks to presence of multifunctional phenolic compounds (mainly taxifolin, catechin and its derivatives) these compositions show potential as antifouling coatings. These phytochemicals provide selective antibacterial activity and improved chemical resistance to non-polar solvents (toluene, acetone). Also, other properties (hardness, adhesion to steel surface, flexural characteristics and impact strength) are promising. In particular, formulations 1 and 6 yield the best composites, which exhibit a good balance of overall parameters.

## Figures and Tables

**Table 1:** Absorbance values of extracts and calculations of TPC.

Extract Name	Abs_765_	TPC [µg GAE/mL]
C120T60	0.9653 ± 0.0047	2674.99 ± 10.61
U30T25	1.0186 ± 0.0065	2795.37 ± 14.68
U15T60	0.9058 ± 0.0039	2540.62 ± 8.81

**Table 2:** Density, TSC, Absorbance and TPC of concentrated extract.

Density [mg/mL]	TSC [%]	Abs_765_	TPC [µg GAE/mL]
0.84 ± 0.04	93.0 ± 1.9	0.765 ± 0.069	5667.6 ± 394.8

**Table 3:** Calculations of percentage share [%] and ratios (PHR and selective) for neat epoxy resin Epidian 5 (Epi5), amine hardener Z-1 (Z-1) and concentrated pine extract (ex). The evaluation of curing progress after one week is presented using colors: blue—reference, completely cured after one week, rigid solid; green—completely cured after one week, rigid solid; yellow—partially cured after one week, soft and sticky solid; orange—too low level of curing, viscous liquid.

	PHR Ratio			Percentage Share [%]			Selective Ratios		
	Epi5	Z-1	ex	Epi5	Z-1	ex	Z-1/Epi5	ex/Epi5	ex/Z-1
0	100	12	0	89.29	10.71	0.00	0.120	0.000	0.000
1	100	8	4	89.29	7.14	3.57	0.080	0.040	0.500
2	100	6	6	89.29	5.36	5.36	0.060	0.060	1.000
3	100	4	8	89.29	3.57	7.14	0.040	0.080	2.000
4	100	3	9	89.29	2.68	8.04	0.030	0.090	3.000
5	100	2	12	87.72	1.75	10.53	0.020	0.120	6.000
6	90	8	14	80.36	7.14	12.50	0.089	0.156	1.750
7	90	6	16	80.36	5.36	14.29	0.067	0.178	2.667
8	90	4	18	80.36	3.57	16.07	0.044	0.200	4.500
9	80	6	26	71.43	5.36	23.21	0.075	0.325	4.333

**Table 4:** Molar quantities of reactive groups and their molar ratios.

	Molar Quantities [mol] Calculated for PHR Ratio			n_H_ of Groups Reacting with Epoxy Ring to Epoxy Groups Molar Ratio
	Epoxy Groups	n_H_ of Amine Groups *	n_H_ of Groups Reacting with Epoxy Ring **	
0	0.480–0.515	0.354–0.492	0.707–0.985	1.37–2.05
1	0.480–0.515	0.236–0.328	0.472–0.656	0.91–1.37
2	0.480–0.515	0.177–0.246	0.354–0.492	0.69–1.03
3	0.480–0.515	0.118–0.164	0.236–0.328	0.46–0.68
4	0.480–0.515	0.088–0.123	0.177–0.246	0.34–0.51
5	0.480–0.515	0.059–0.082	0.118–0.164	0.23–0.34
6	0.432–0.464	0.236–0.328	0.472–0.656	1.02–1.52
7	0.432–0.464	0.177–0.246	0.354–0.492	0.76–1.14
8	0.432–0.464	0.118–0.164	0.236–0.328	0.51–0.76
9	0.384–0.412	0.177–0.246	0.354–0.492	0.86–1.28

*—minimum n_H_ of amine groups is less likely according to MSDS of Z-1 hardener, which is pure TETA; **—summary of n_H_ of amine groups and n_H_ of secondary hydroxyl groups from epoxy-amine reactions (2 × n_H_ of amine groups).

**Table 5:** Specific enthalpy calculations.

Composition	Enthalpy [J]	Weight of Sample [mg]	Specific Enthalpy [J/g]
0	−14.640	49.0	−298.78
1	−5.880	47.0	−125.11
2	−4.120	38.6	−106.74
3	−0.517	32.3	−16.01
6	−6.240	49.0	−127.35
7	−5.420	40.8	−132.84
8	−5.510	39.3	−140.20
9	−4.490	36.3	−123.69

**Table 6:** Antibacterial activity (expressed as R values) of epoxy composites with extract against selected bacteria species.

	Composition					
Bacteria Species	0	1	2	6	7	9
*Escherichia coli*	−1.24	−1.21	−0.37	−0.41	−0.91	3.53
*Staphylococcus aureus*	−0.12	−0.08	−0.08	0.50	0.24	4.92
*Bacillus subtilis*	−0.19	0.60	−0.35	0.83	0.30	3.20
*Enterococcus faecium*	5.00	0.27	0.34	1.83	−0.14	5.00
*Klebsiella aerogenes*	−0.33	−0.74	−0.57	−0.80	−1.00	5.34

**Table 7:** Results of adhesion tests.

Composition	Cross Cut Adhesion Test	X-Cut Adhesion Test
0	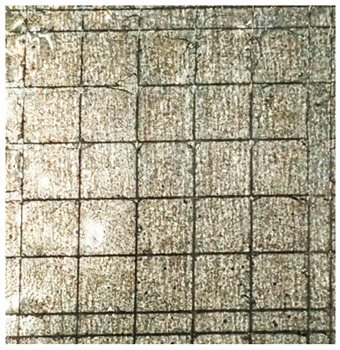 Rating: 1	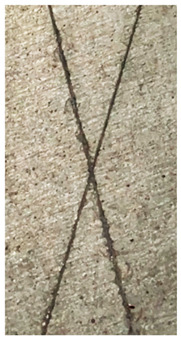 Rating: 0
1	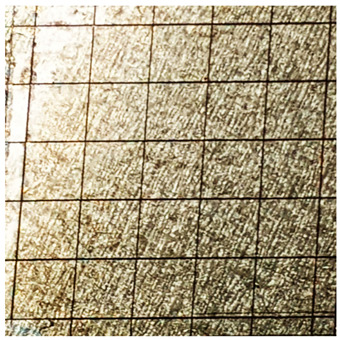 Rating: 0	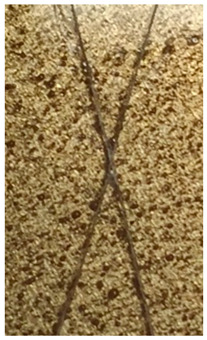 Rating: 0
2	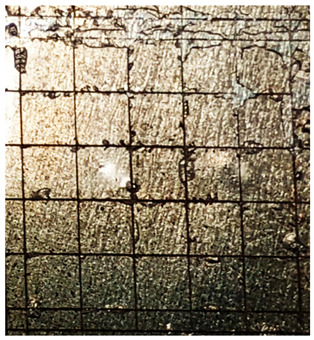 Rating: 1	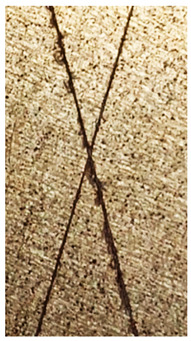 Rating: 1
6	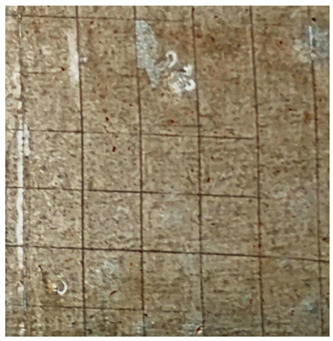 Rating: 2	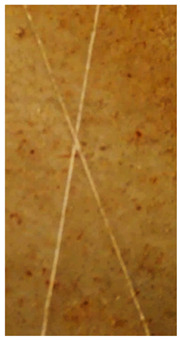 Rating: 0
7	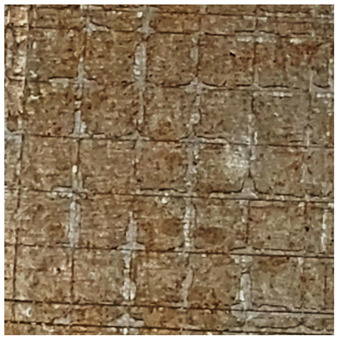 Rating: 2	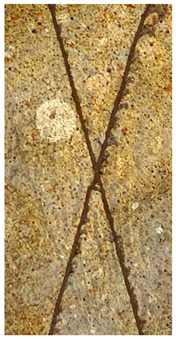 Rating: 1
9	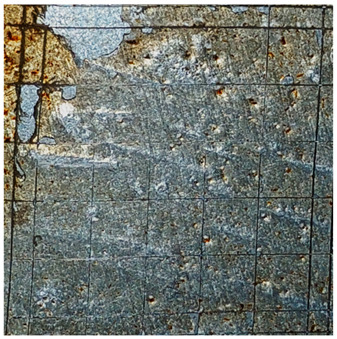 Rating: 2	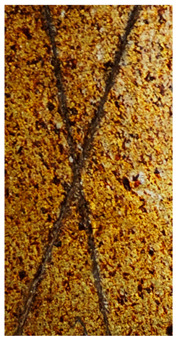 Rating: 1

**Table 8:** List of extracts for qualitative and quantitative analysis.

Extract Name	Type of Extraction	Temperature [°C]	Extraction Time [min]
C120T60	conventional	60	120
U30T25	ultrasound-assisted	25	30
U15T60	ultrasound-assisted	60	15

**Table 9:** Regime of HPLC separation.

Time [min]	% of Phase A (Formic Acid 5% Water Solution)	% of Phase B (Acetonitrile)	Flow [mL/min]
0	97	3	0.8
2	97	3	0.8
15	85	15	0.8
24	82	18	0.8
55	75	25	0.8
60	97	3	0.8

**Table 10:** Epoxy compositions formulations (expressed as PHR).

	Number:	0	1	2	3	4	5	6	7	8	9
Component	Epidian 5	100	100	100	100	100	100	90	90	90	80
	Z-1	12	8	6	4	3	2	8	6	4	6
	extract	0	4	6	8	9	10	14	16	18	26

## Data Availability

The data presented in this study are mostly openly available. DOI addresses are provided in the References Section.
